# Supplementary material for: Characterizing steroid hormone receptor chromatin binding landscapes in male and female breast cancer
Source: Nat Commun. 2018 Feb 2;9:482. doi: 10.1038/s41467-018-02856-2 (PMC5797120; doi:10.1038/s41467-018-02856-2)
Supplement: Supplementary file 1 — Supplementary Information [file 41467_2018_2856_MOESM1_ESM.pdf]

1

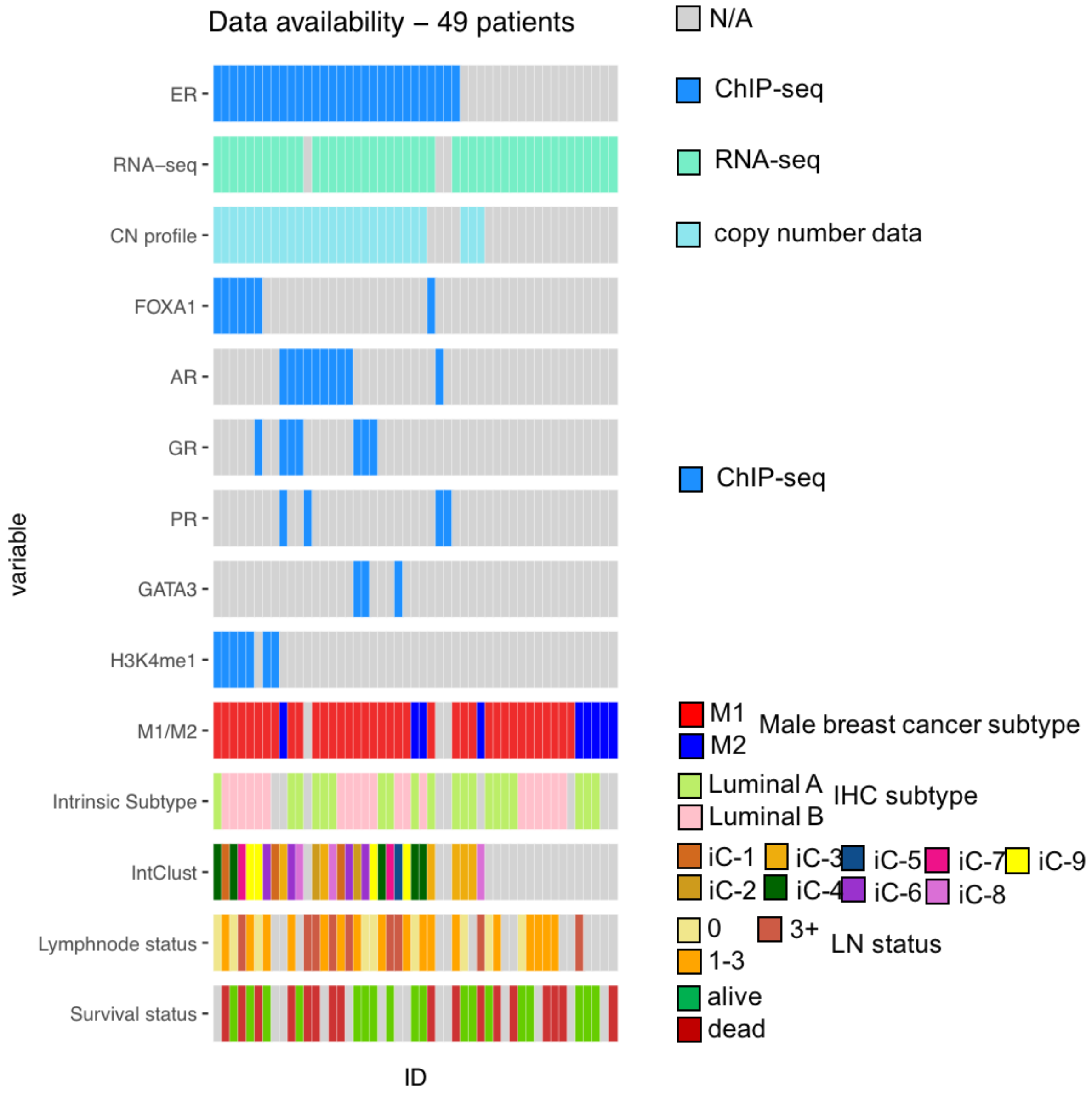

Supplementary Figure 1: Color map depicting the data collected from male breast tumors.

Available data for ChIP-sequencing, RNA-sequencing, DNA copy number data, male breast cancer intrinsic subtypes (M1/M2), IHC-derived intrinsic subtypes, IntClust analyses, lymph node status and survival data are indicated for each patient.

2

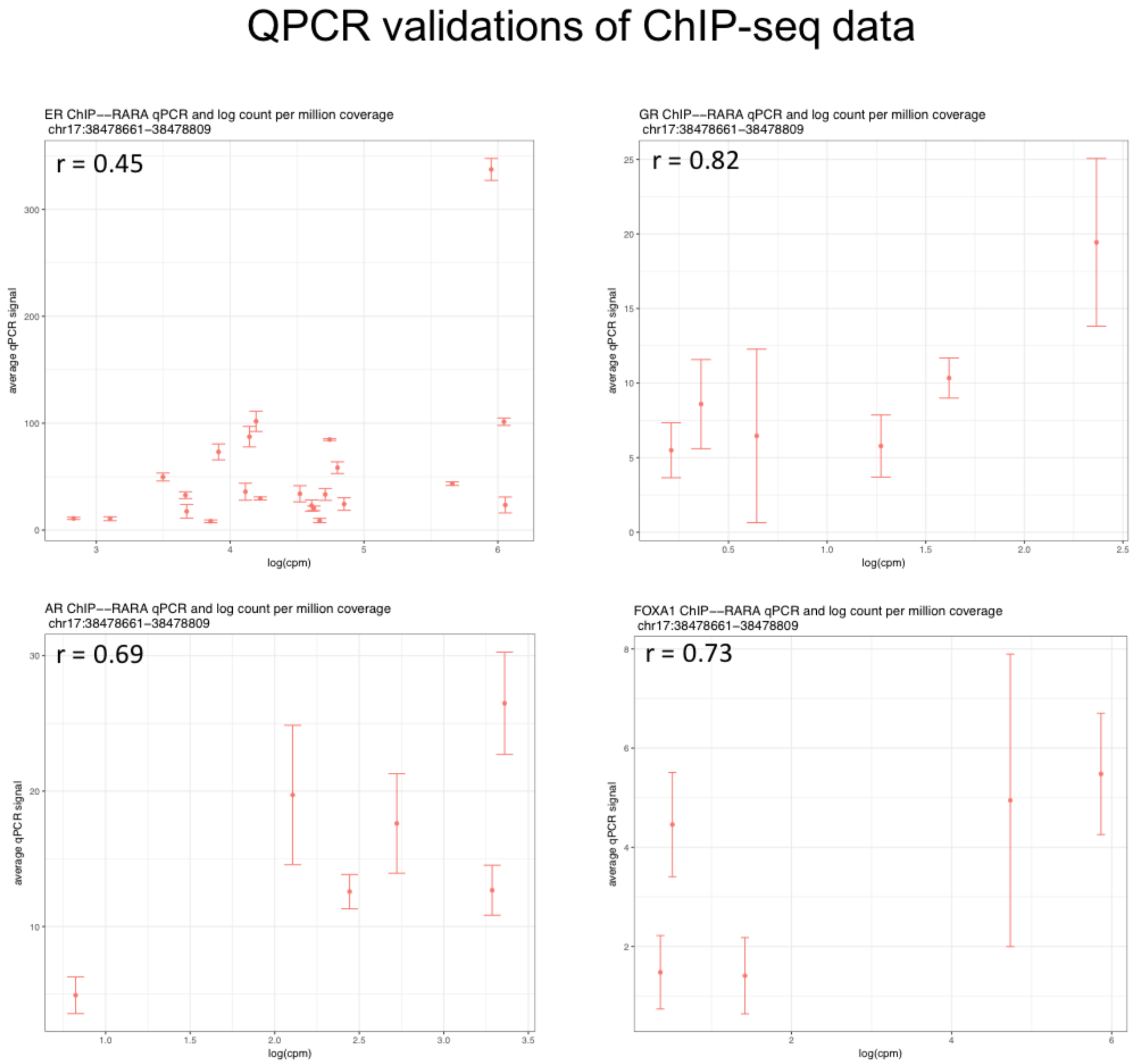

Supplementary Figure 2: ChIP-QPCR validations of ChIP-seq data for ER, AR, FOXA1 and GR.

ChIP was performed for ER, AR, GR and FOXA1, after which sample was split in two. Part of the sample was processed for library preparation and sequenced, while remaining sample was used for QPCR analyses. Graphs show correlations between ChIP-QPCR enrichment at RARA enhancer, in relation to log-transformed count per million (CPM) ChIP-seq data at the same genomic locus. Error bars show standard deviations from 3 measurements. Pearson correlation coefficient is shown.

3

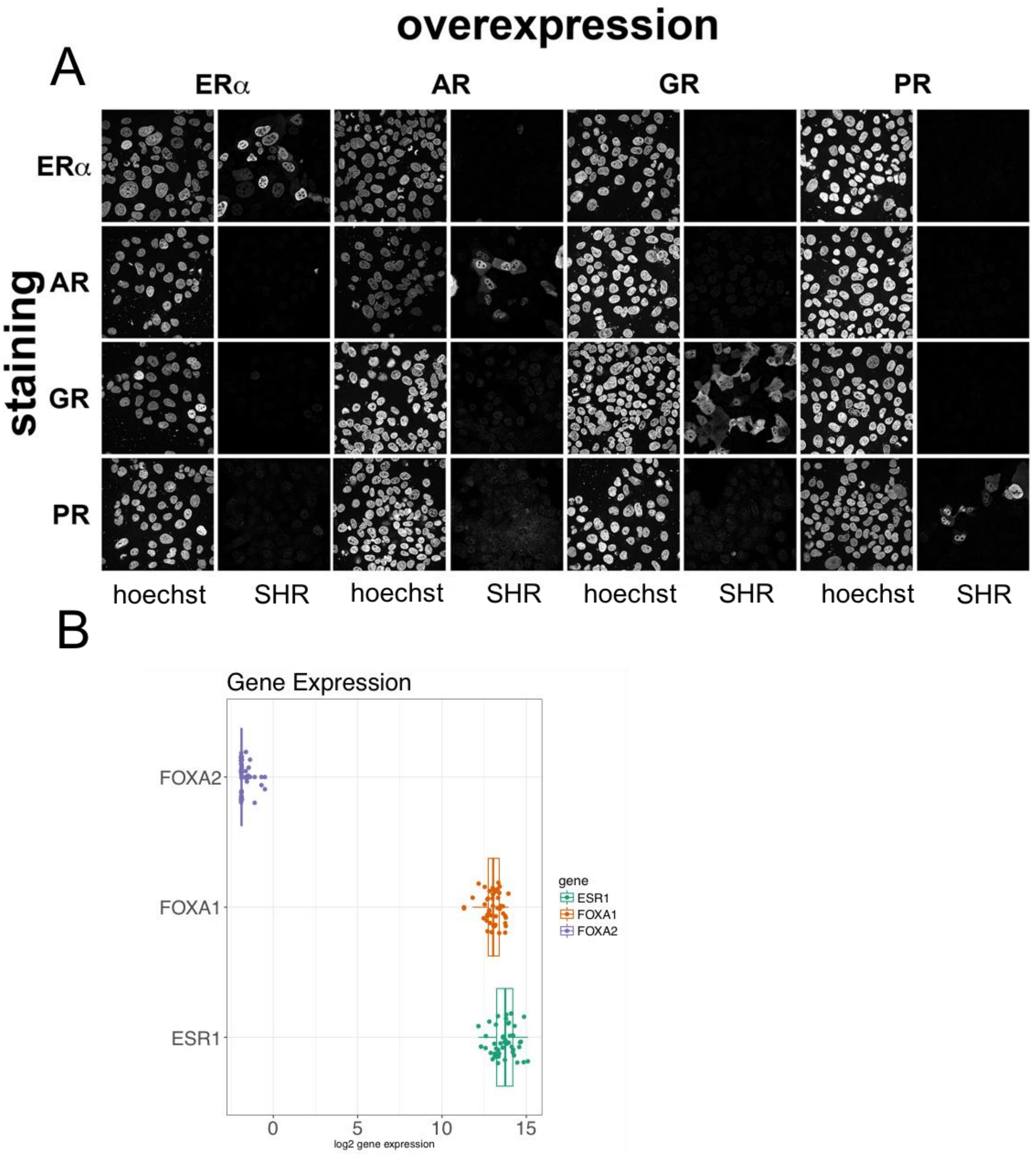

Supplementary Figure 3: **Validation of antibodies used in ChIP-seq**

A. U2OS cells were transiently transfected with vectors encoding for ERα, AR, GR or PR, fixed with 3,7% PFA and processed for immunofluorensence staininig. Only slides stained positive when antibody was used for the overexpression hormone receptor. Hoechst: DNA staining; SHR: steroid hormone receptor staining.
B. RNA-seq derived gene expression (log2) for ESR1, FOXA1 and FOXA2 in the male breast cancer samples. Even though the FOXA1 antibody detects both FOXA1 and FOXA2, only FOXA1 is expressed.

4

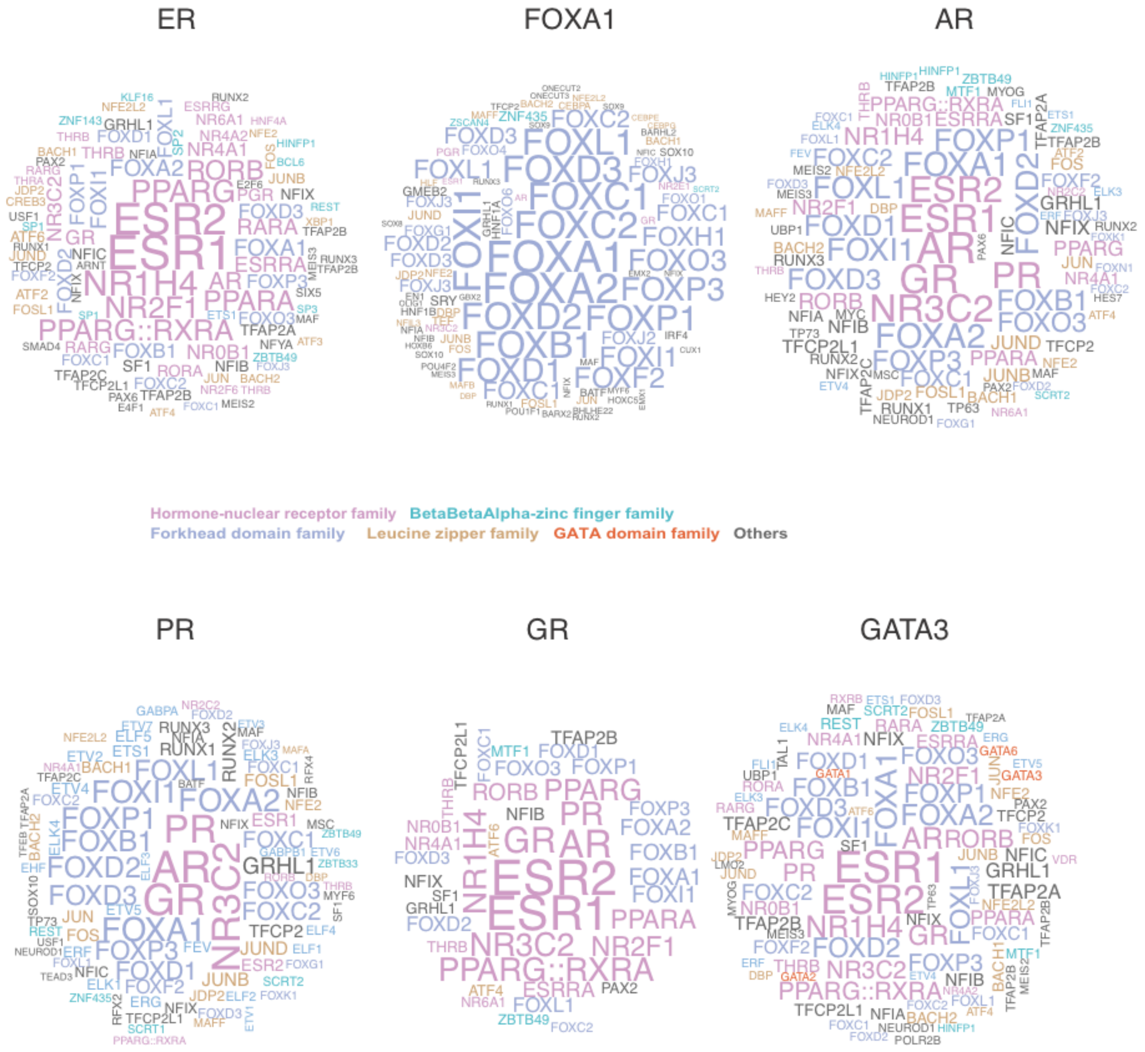

Supplementary Figure 4: Enriched sequence motifs for ChIP-seq on ERα, FOXA1, AR, PR, GR and GATA3.

Wordclouds showing enriched sequence motifs for ChIP-seq on ERα, FOXA1, AR, PR, GR and GATA3. Font size is proportional to significance of motif enrichment (Z-score), and font color represents the family of motifs.

5

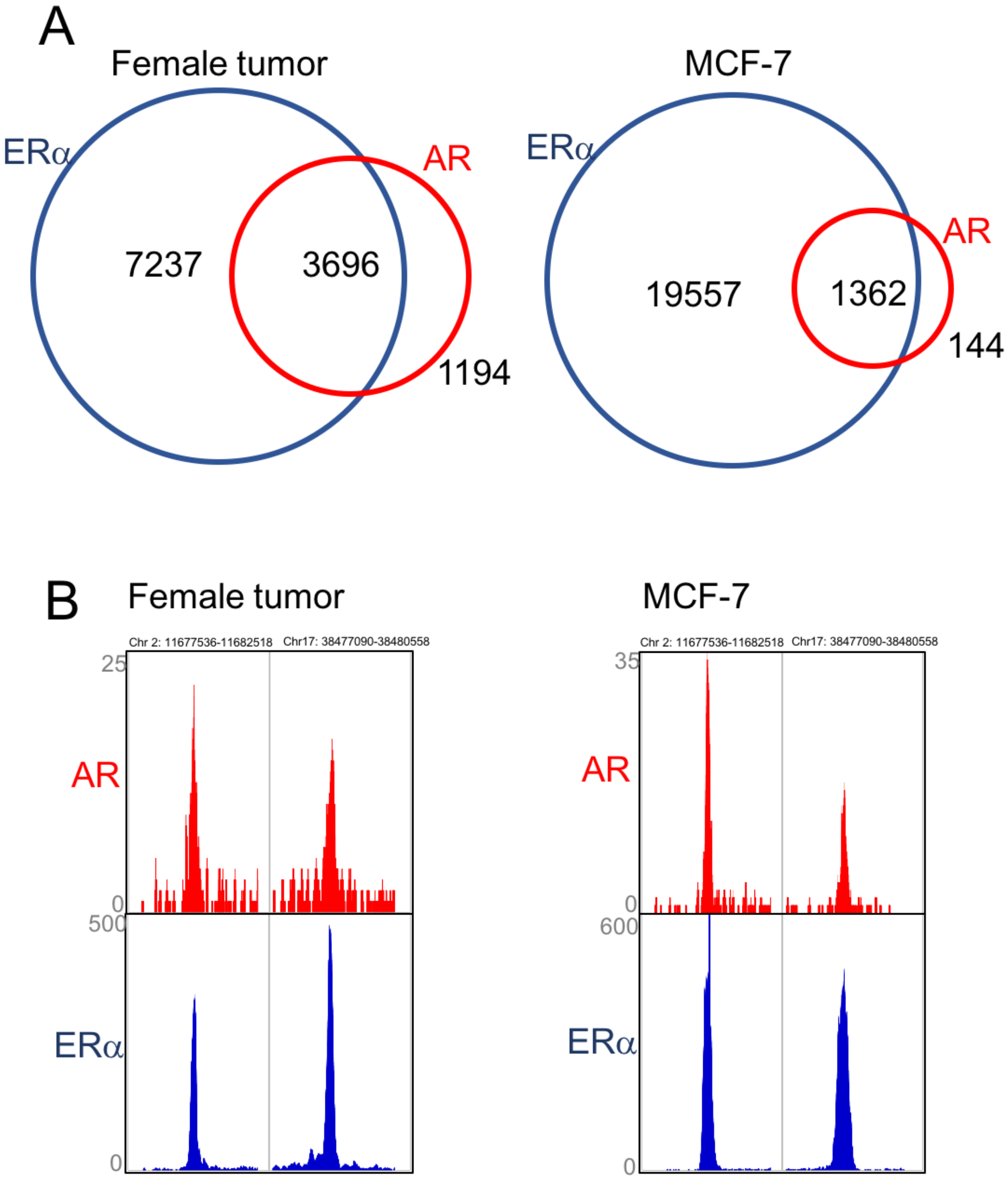

Supplementary Figure 5: **Overlap of ERα and AR binding in a female breast tumor and MCF-7 cells**

A. Venn diagram, showing shared and unique sites of ERα and AR in an ER+ female breast tumor (left) and MCF-7 cells (right).
B. Genome browser snapshots, showing shared peaks for ERα and AR in an ER+ breast tumor (left) and MCF-7 cells (right). Read count and genomic coordinates are indicated.

6

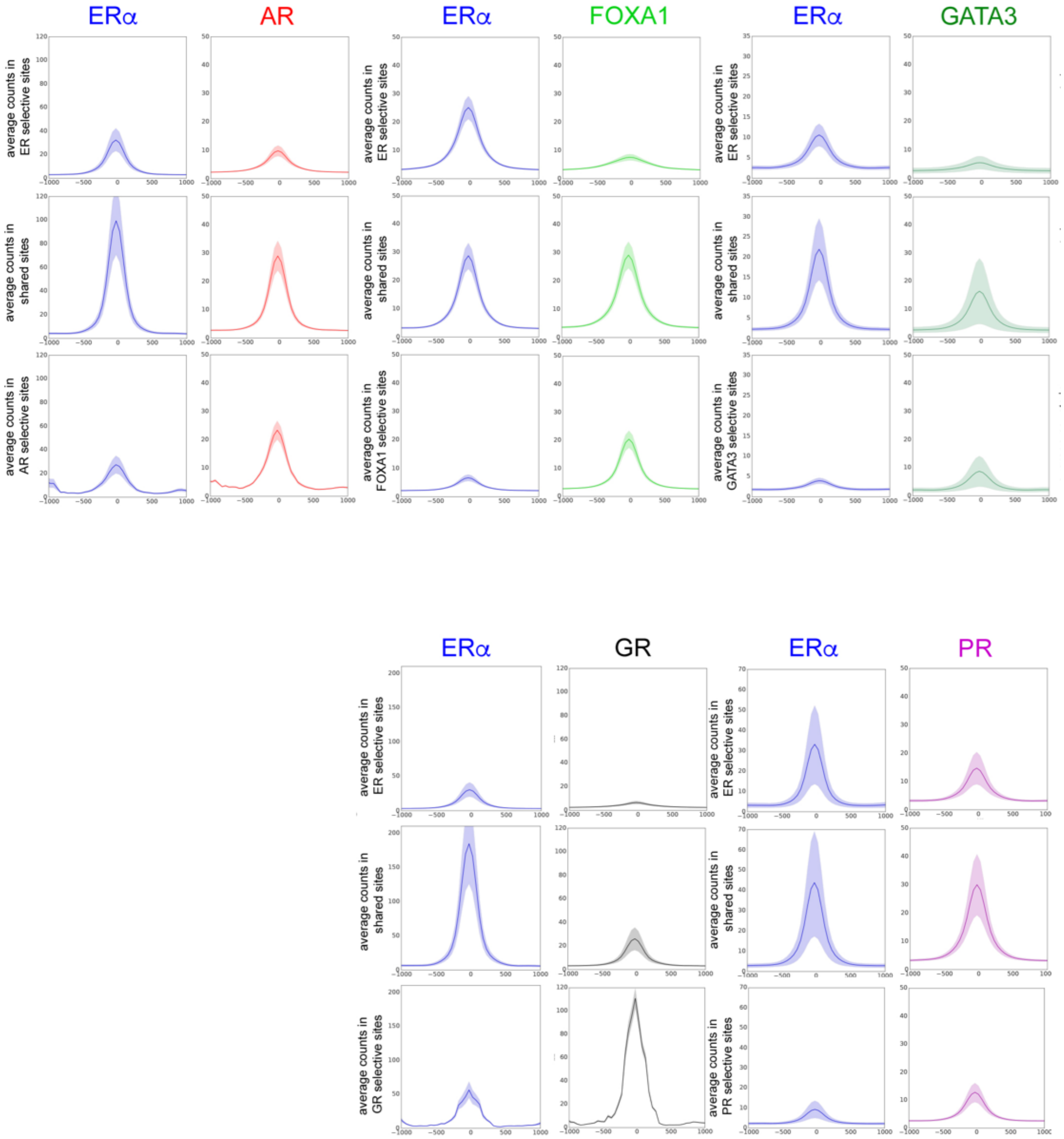

Supplementary Figure 6: **Average read count at shared and selective sites between factors.**

Average read count profiles within +/- 1kb from the center of binding sites defined by Venn diagram analysis in Figure 2C. 75% confidence interval of average profile indicated with shading.

7

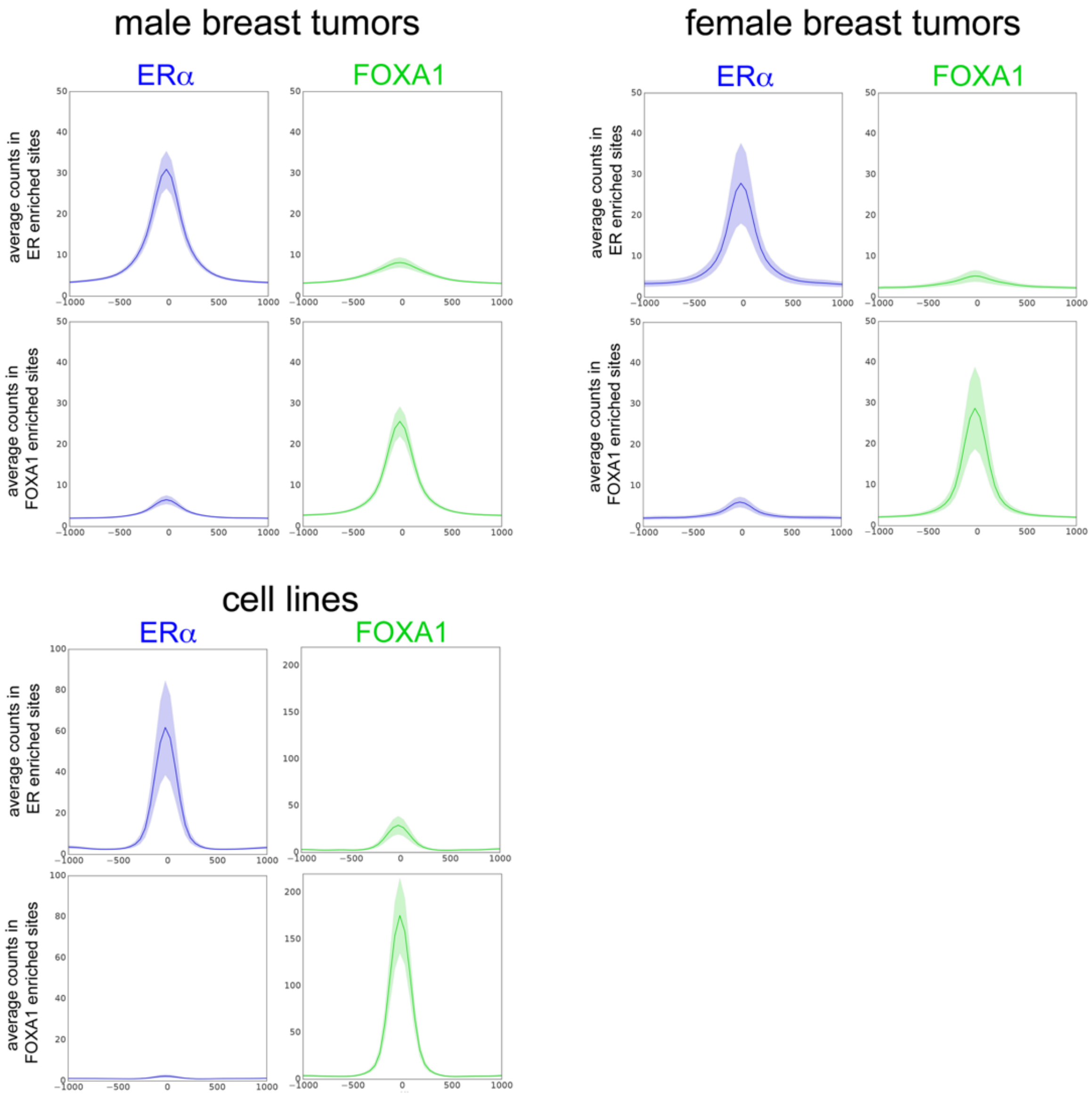

Supplementary Figure 7: Average readcount of ERα and FOXA1 at differentially enriched binding sites in male breast tumors, female breast tumors and cell lines.

Average read count profiles within +/- 1kb from the center of sites enriched for ERα and FOXA1. 75% confidence interval of average profile indicated with shading.

8

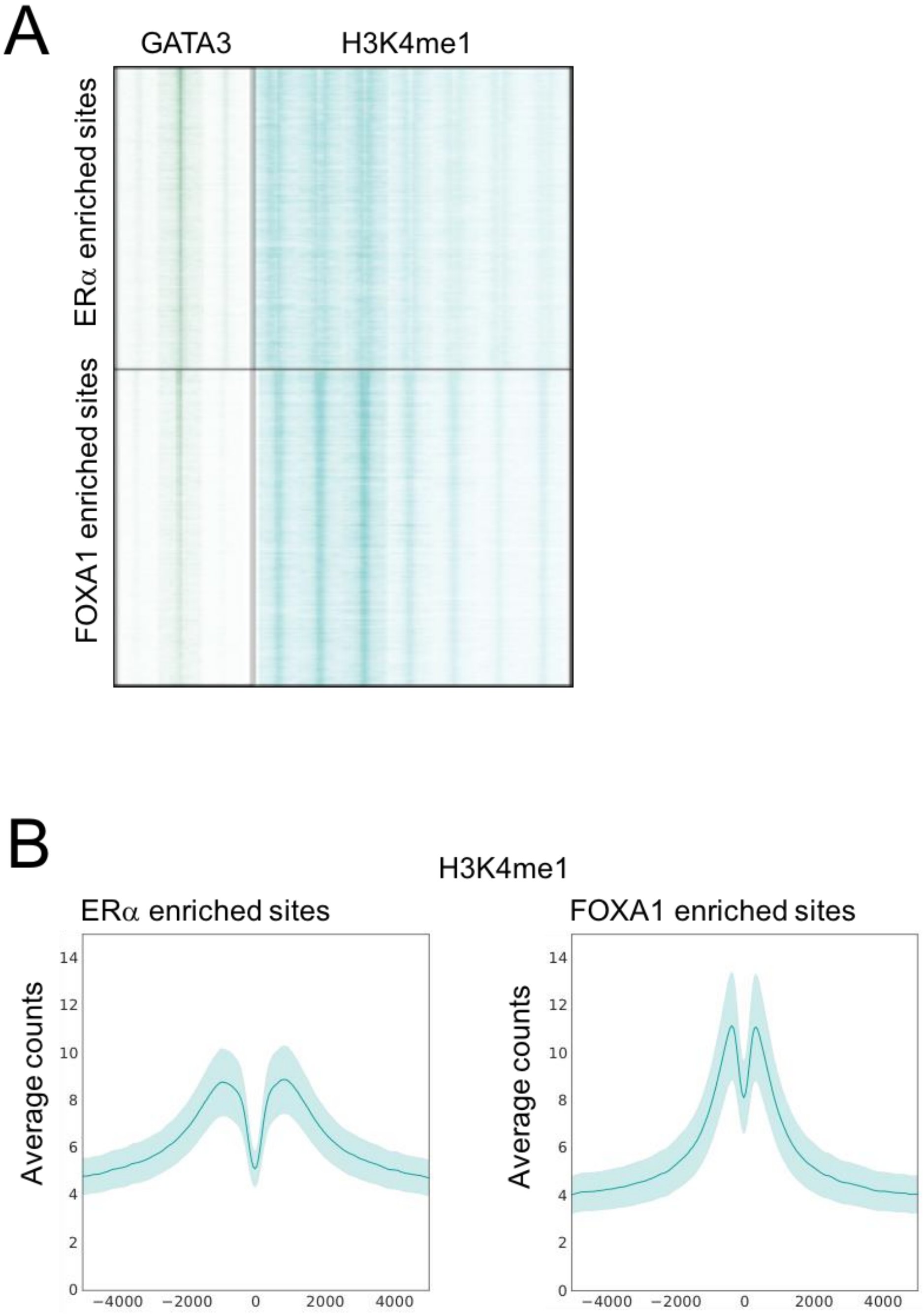

Supplementary Figure 8: **ERα selective sites devoid of FOXA1 are enriched at promoter regions devoid of enhancer-mark H3K4me1.**

A. Heatmap visualization of GATA3 (green) and H3K4me1 (cyan) signal at sites differentially enriched between ERα and FOXA1.
B. H3K4me1 read count profile in sites enriched for ERα or FOXA1. 75% confidence interval of average profile indicated with shading.

9

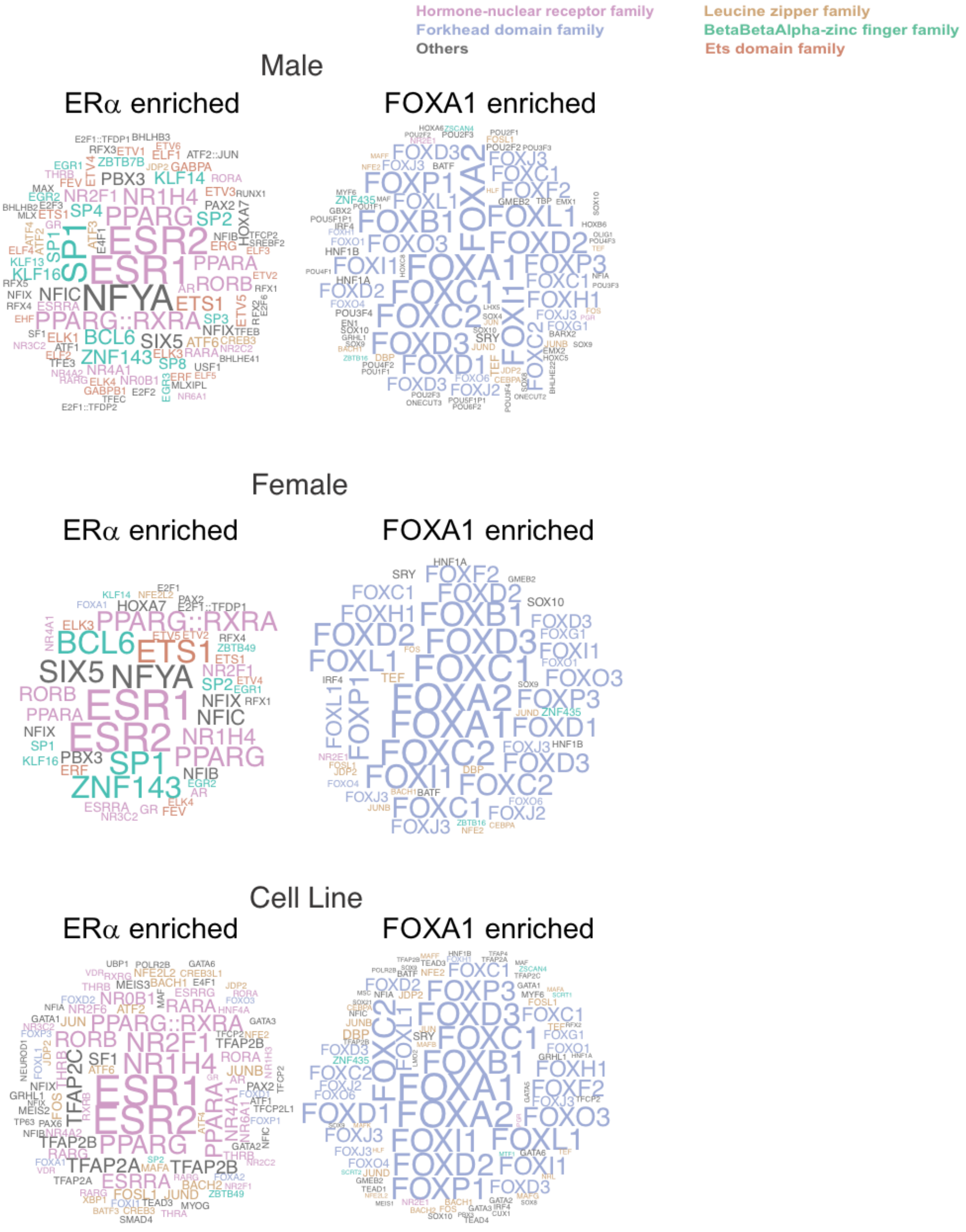

Supplementary Figure 9: Enriched sequence motifs for genomic regions differentially enriched between ERα and FOXA1 in male breast cancers, female breast cancers and cell lines.

Wordclouds showing top sequence motifs for sites differentially enriched between ERα and FOXA1. Font size is proportional to significance of motif enrichment (Z-score), and font color represents the family of motifs.

10

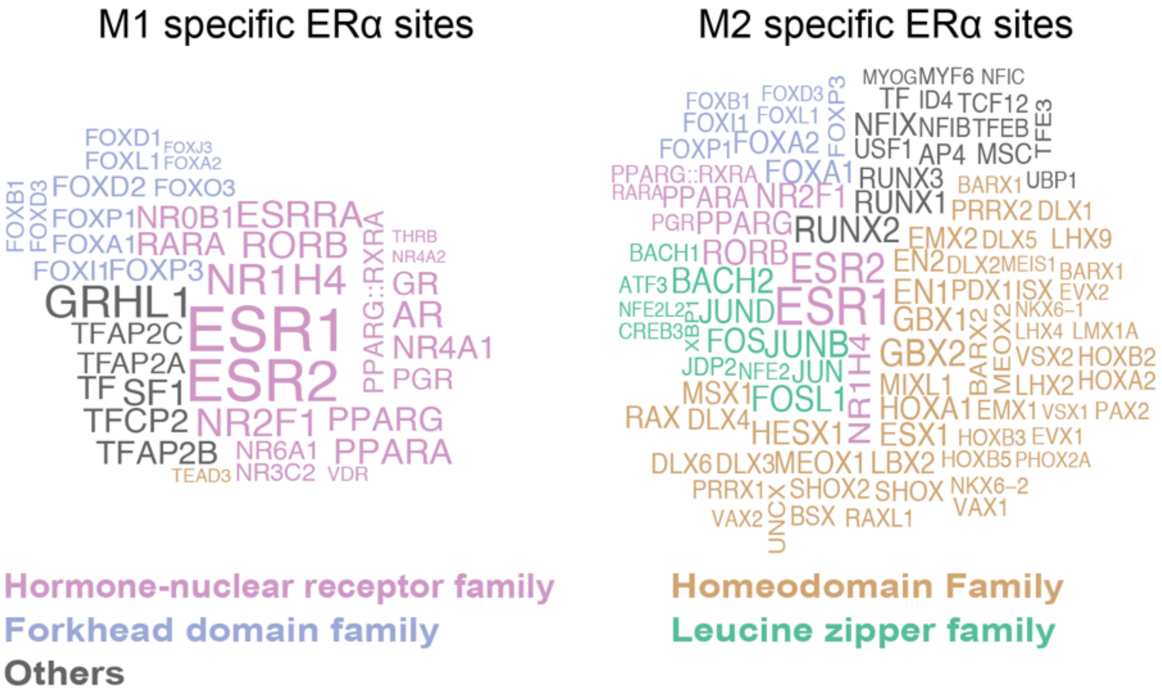

Supplementary Figure 10: **Enriched sequence motifs for M1 and M2 specific ERα binding sites.**

Wordclouds showing top sequence motifs for M1 and M2 specific ERα sites. Font size is proportional to significance of motif enrichment (Z-score), and font color represents the family of motifs.

11

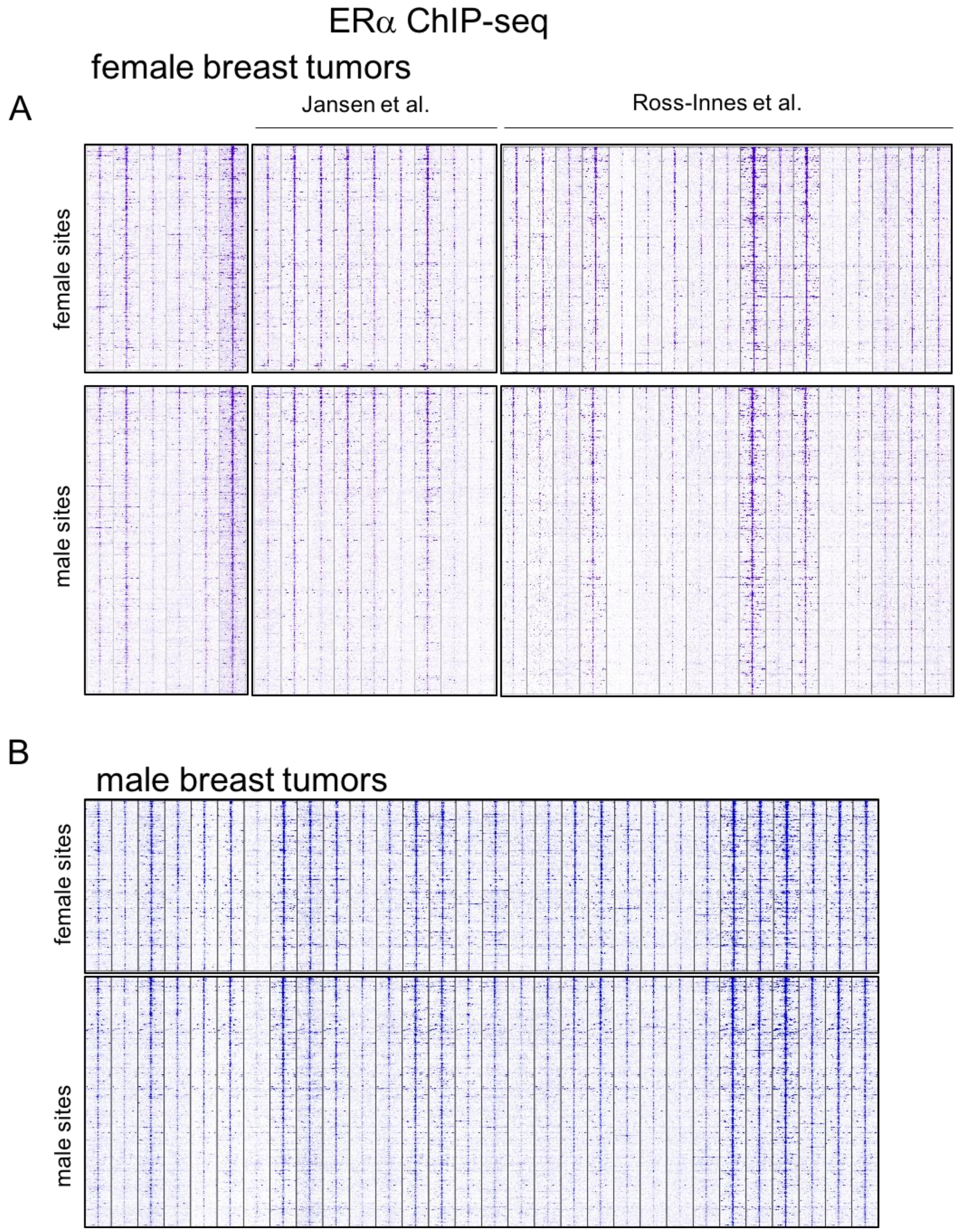

Supplementary Figure 11: **ERα binding shared between genders**

A. Heatmaps, separately depicting ERα ChIP-seq data in female breast tumors generated in this study, or from publicly available dataseries, Jansen et al. and Ross-Innes et al. Binding sites identified in female tumors (top) and male tumors (bottom) are depicted separately.
B. As in A., but now male breast cancer ChIP-seq data is used.

12

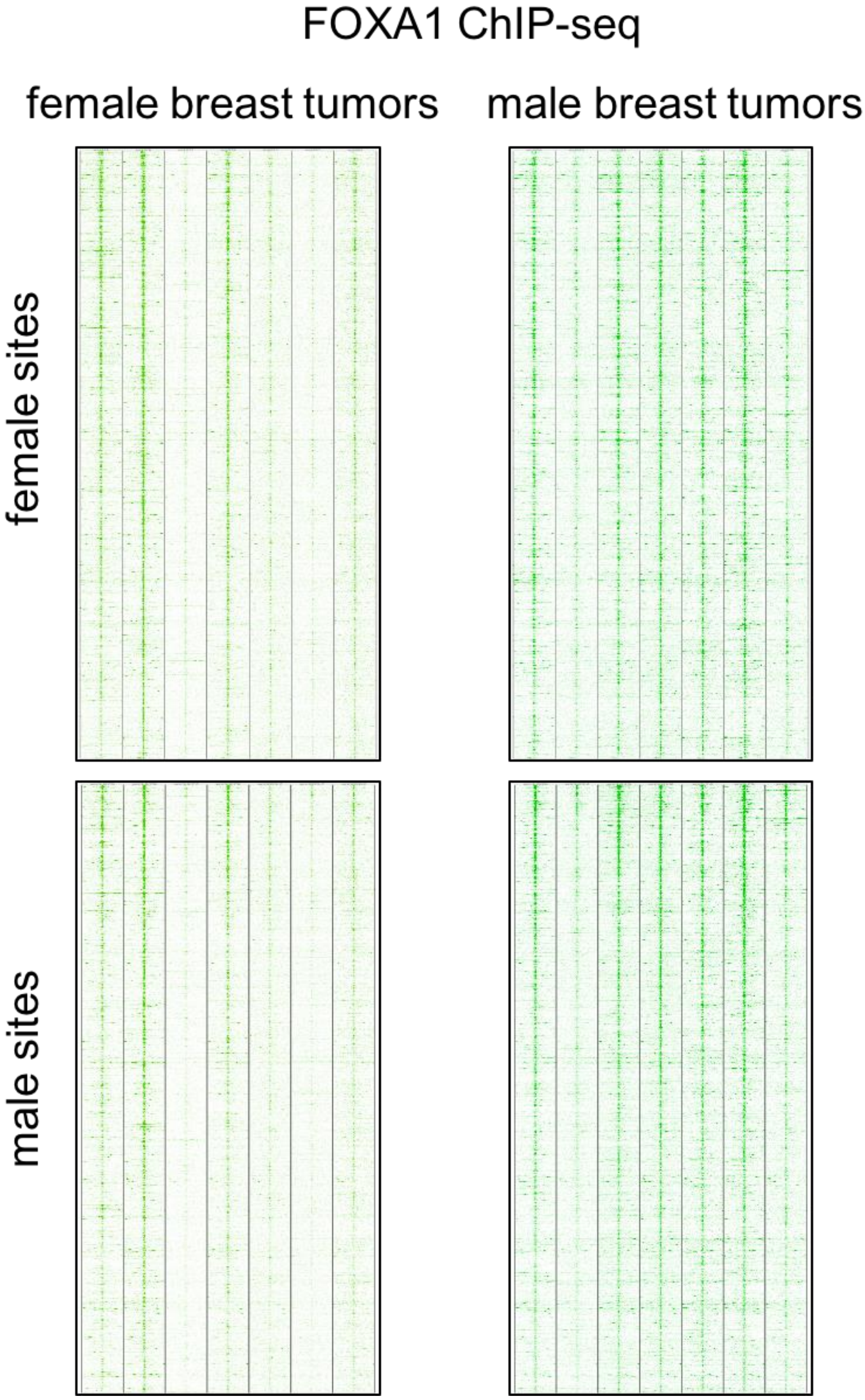

Supplementary Figure 12: **FOXA1 binding shared between genders**

Heatmaps, depicting FOXA1 ChIP-seq data in female breast tumors (left) or male breast tumors (right). Binding sites identified in female tumors (top) and male tumors (bottom) are depicted separately.

13

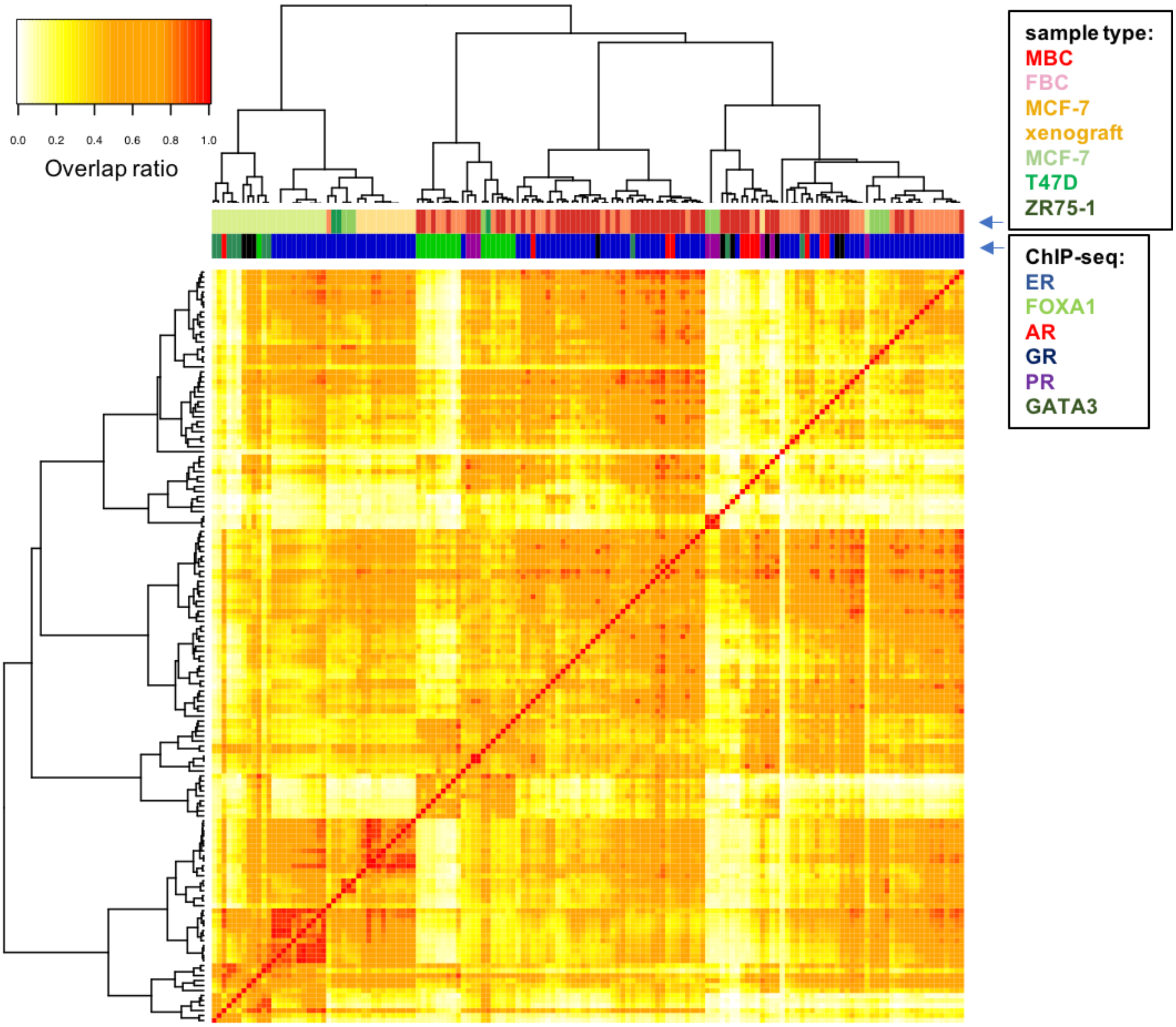

Supplementary Figure 13: **Clustering analysis of ERα, AR, FOXA1, GATA3, GR and PR binding sites between genders and cell lines**

Clustering analysis of ChIP-seq data for ERα, AR, FOXA1, GATA3, GR and PR, where colorscale indicates level of overlap for each pair of ChIP-seq datasets. Top color labels indicate female breast cancers (pink), male breast cancers (red), MCF-7 xenografts (yellow) and in vitro cell line cultures MCF-7 (lime), T47D (light green) and ZR751 (dark green). Bottom color labels indicate ChIP-seq for ERa(blue), FOXA1 (light green), AR (red), GATA3 (dark green), GR (black), and PR (purple).

14

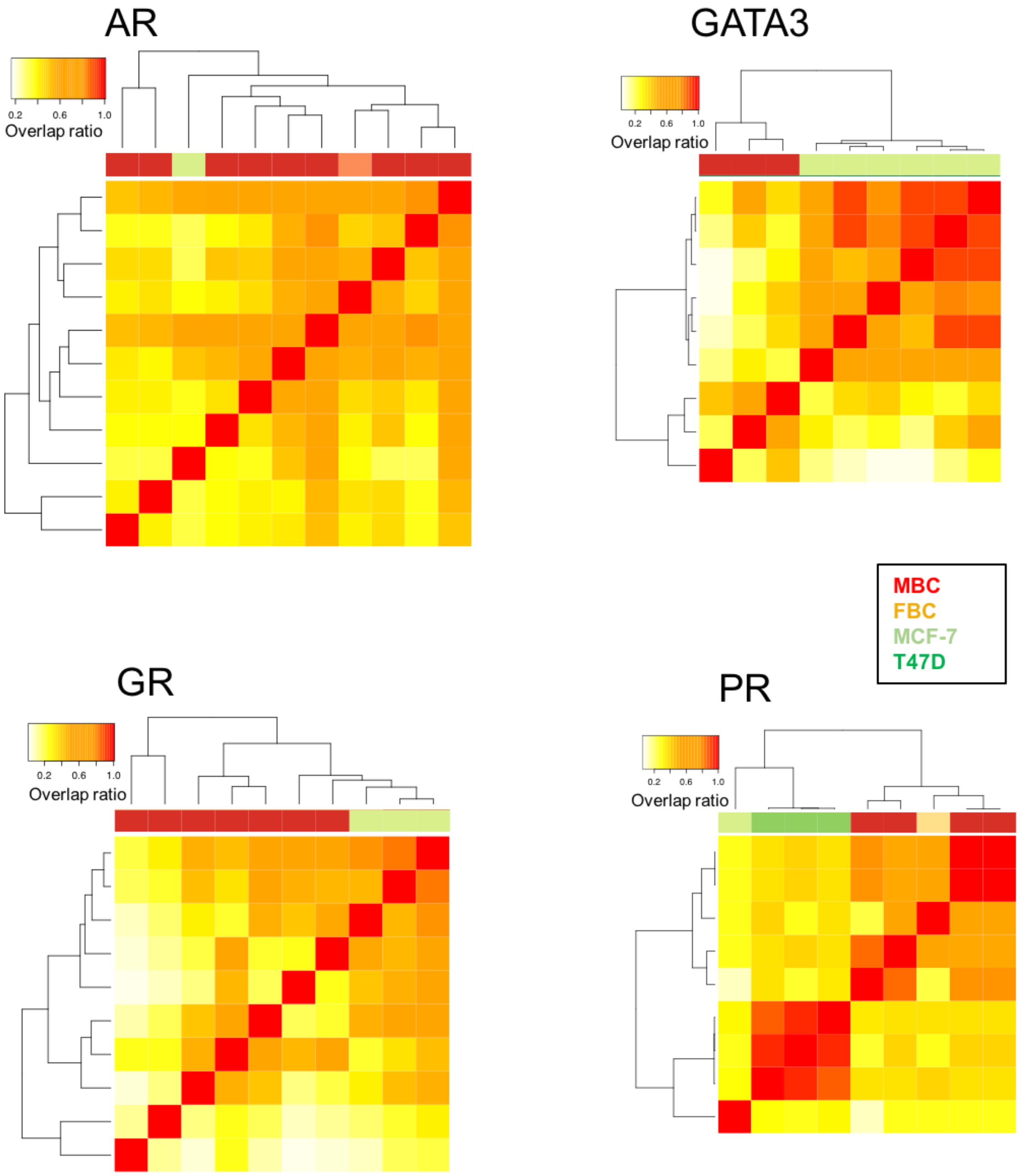

Supplementary Figure 14: Clustering analysis of AR, GATA3, GR and PR binding sites between genders and cell lines

Separate clustering analysis of ChIP-seq data for AR, GATA3, GR and PR, where colorscale indicates overlap ratio between a pair of samples. Color labels indicate female breast cancers (pink), male breast cancers (red) and in vitro cell line cultures MCF-7 (lime) and T47D (light green).

15

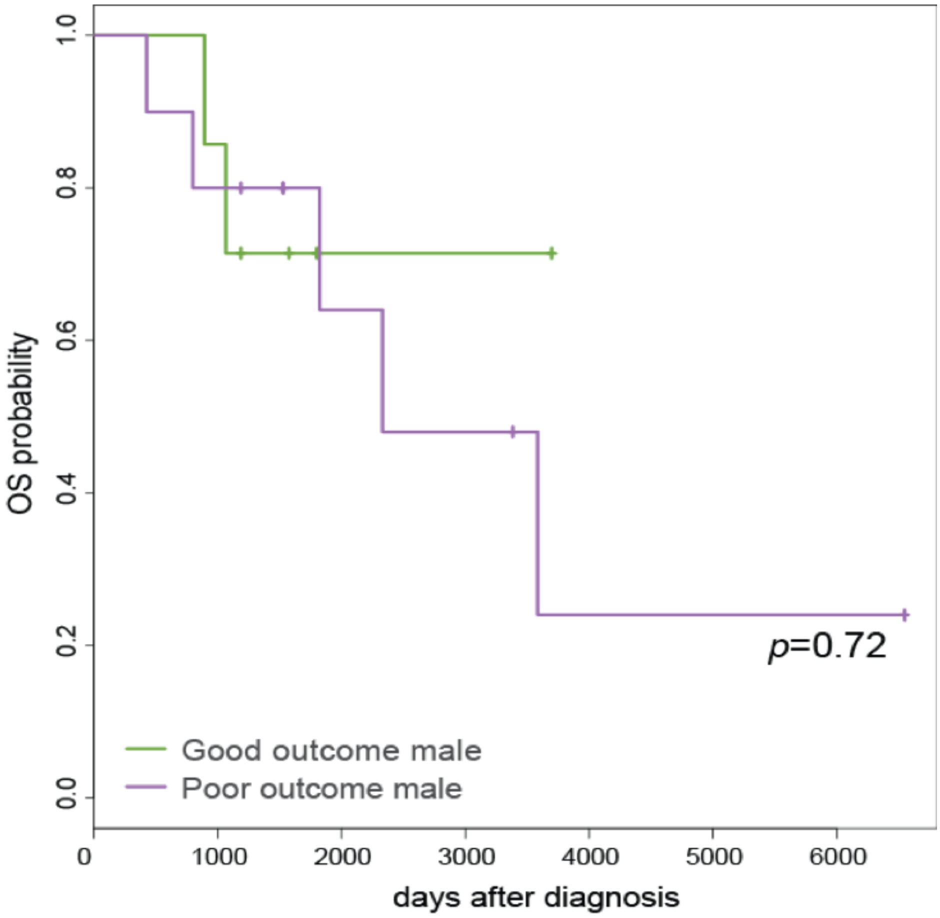

Supplementary Figure 15: **ERα binding sites that can classify female outcome did not show predictive potential in male.**

Kaplan-Meier plot showing overall survival of good outcome (green) and poor outcome (purple) male patients, where outcome is predicted based on ERα binding profiles in the sites that can classify female outcome. 17 patients with both known survival status and ERα ChIP-seq data are used for the analysis. Log-rank p-value is indicated in the plot. Overall Survival (OS) probability and days after diagnosis are indicated in y- and x-axis, respectively.

16

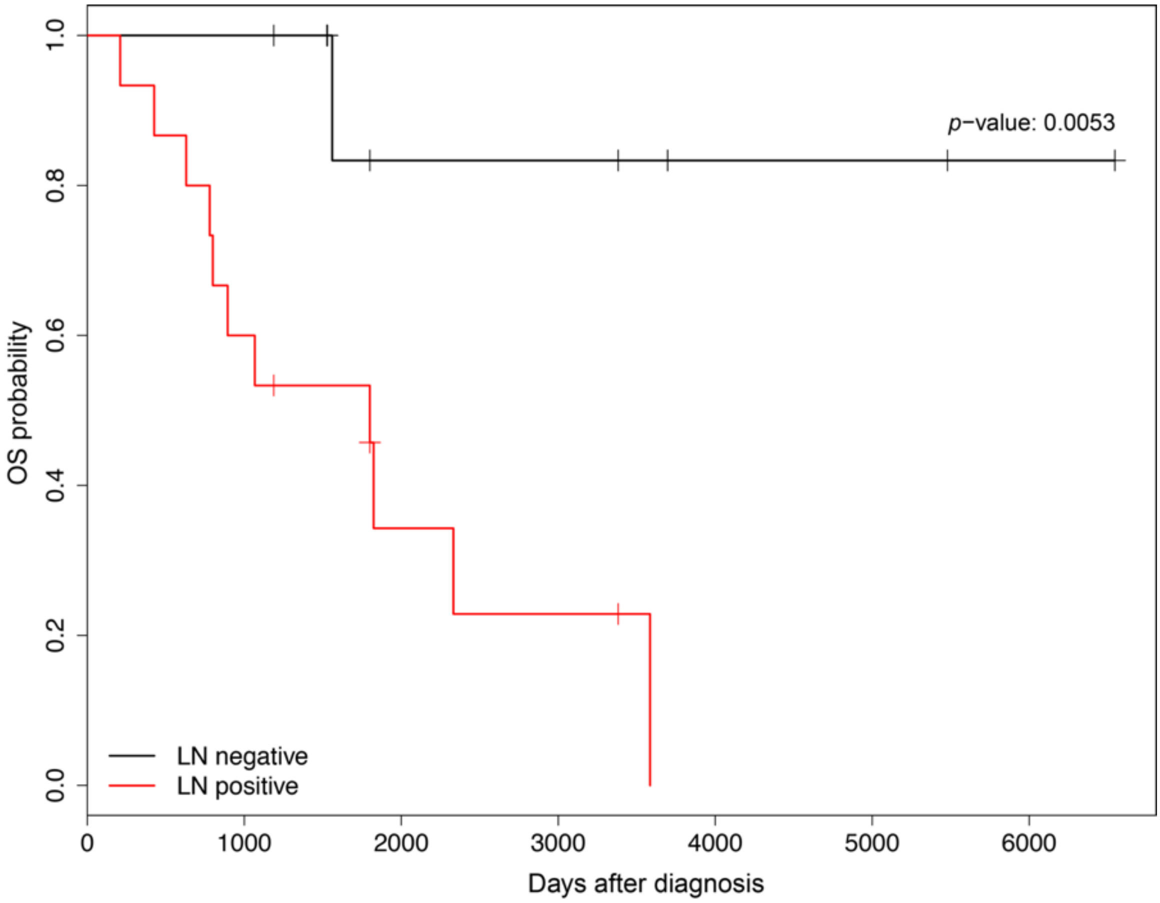

Supplementary Figure 16: **LN status significantly predict patient outcome.**

Kaplan-Meier plot showing overall survival of LN-negative (black) and positive (red) male patients. Log-rank p-value is indicated in the plot. Overall Survival (OS) probability and days after diagnosis are indicated in y- and x-axis, respectively.

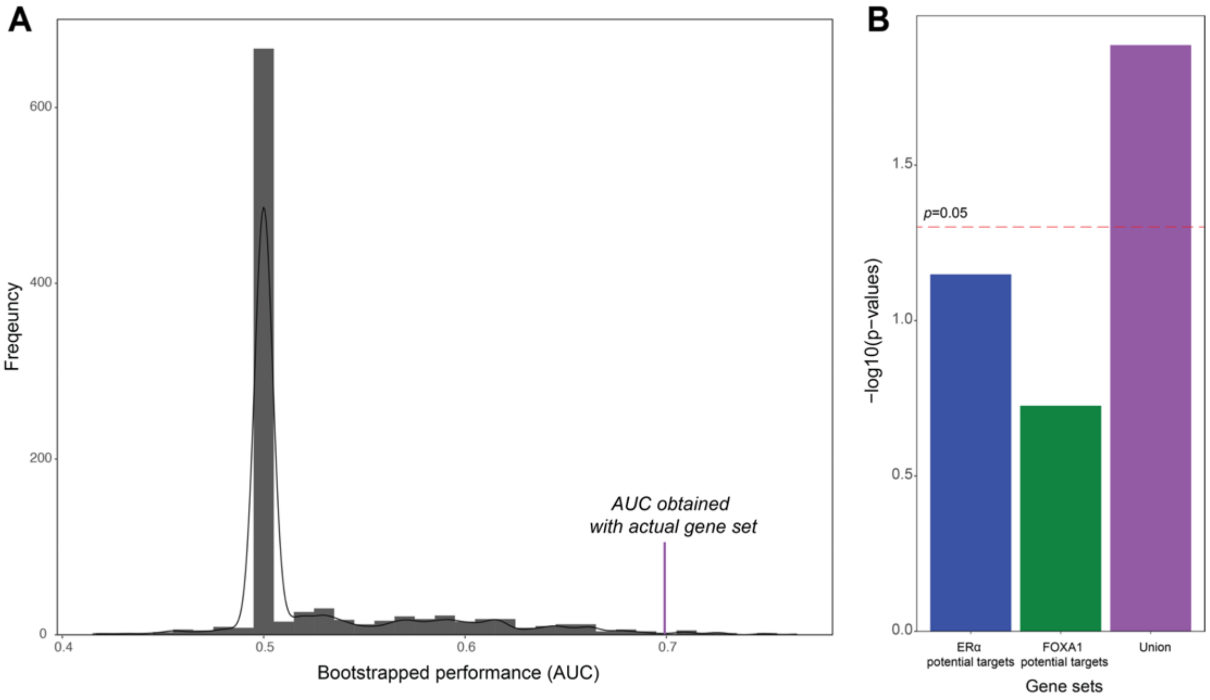

Supplementary Figure 17: **Significance assessment of the gene expression signature to predict patient outcome using bootstrapping analysis.**

A. Distribution of AUC performance obtained from 1000 model trained with bootstrapped gene sets. The size of gene sets is fixed to the number of unions of potential targets. Performance obtained with union of potential target genes is indicated with a purple line.
B. Bar plot indicating significance of model performance compared with bootstrapped AUC distribution for ERα (blue), FOXA1 (green) and union (purple) potential targets. The significance measure is –log10 transformed *p*-value, and a dotted red line indicates p value of 0.05.

**Supplementary Table 1. Male Patient Characteristics**				
Variable	M1<br>N = 37	M2<br>N = 9	Missing Data<br>N = 3	*P*
Age at diagnosis				0.90[a]
Mean	65.2	65.3	82	
Range	36 - 81	35 - 86	NA	
Tumor size (cm)				< 0.05[a]
Missing data	5	1	0	
0-1	0	0	0	
>1-2	8	6	2	
>2-5	24	2	1	
>5	0	0	0	
Mean	2.5	1.7	NA	
OS status				0.41[b]
Missing data	10	2	2	
Alive	13	5	0	
Dead	14	2	1	
LN status				0.61[b]
Missing data	6	4	2	
Positive	23	3	1	
Negative	8	2	0	
ER status				0.19[b]
Positive	35	7	1	
Negative	0	1	0	
PR status				0.30[b]
Positive	27	8	1	
Negative	8	0	0	
AR status				1.0[b]
Positive	33	5	0	
Negative	1	0	0	
GR status				1.0[b]
Positive	12	1	0	
Negative	2	0	0	
FOXA1 status				< 0.05[b]
Positive	14	1	0	
Negative	0	0	0	
GATA3 status				< 0.05[b]
Positive	14	1	0	
Negative	0	0	0	
Breast surgery				0.18[b]
Missing data	16	1	2	
Breast conserving	4	4	1	
Modified radical mastectomy	4	0	14	
Radical mastectomy	3	0	0	
Adj. endocrine therapy				1.0[b]
Missing data	16	1	2	
No treatment	8	3	0	
Treatment	13	5	1	
Adj. chemotherapy				1.0 [b]
Missing data	16	1	2	
No treatment	18	7	1	
Treatment	3	1	0	
Adj. radiotherapy				< 0.05[b]
Missing data	16	1	2	
No treatment	4	6	0	
Treatment	17	2	1	

Abbreviations: Adj., adjuvant; LN, lymphnode; OS, overall survival; a=Wilcoxon rank sum test; b=Pearson Chi-square test with simulated P-value based on 2000 replicates; missing data = unable to obtain clinical or molecular data

Supplementary Table 2. Female Patient Characteristics

Sample	ER	PR	HER2	Age	LN metastasis	Year collection	Tumor size (cm)	Tumor grade
1	100	30	negative	53	FALSE	2011	NA	2
2	100	100	negative	73	FALSE	2011	NA	2
3	100	100	negative	59	FALSE	2012	NA	1
4	90	30	positive	56	TRUE	2011	4.2	3
5	95	15	negative	35	FALSE	2013	1.8	2
6	100	0	negative	50	TRUE	2009	1.8	2
7	100	40	negative	63	FALSE	2013	1.5	2
8	100	40	negative	64	NA	NA	3.5	3

Supplementary Table 3. Publicly available ChIP-seq datasets used in this study

SampleID	DataSeries	Factor	Tissue	Condition
GSM631471	GSE25710	FOXA1	MCF7	FullMedia
GSM631472	GSE25710	FOXA1	ZR751	FullMedia
GSM631473	GSE25710	FOXA1	T47D	FullMedia
GSM631480/GSM631481/GSM631482	GSE25710	ER	MCF7	FullMedia
GSM631483/GSM631484	GSE25710	ER	MCF7	FullMedia
GSM631485/GSM631486	GSE25710	ER	ZR751	FullMedia
GSM631487/GSM631488	GSE25710	ER	ZR751	FullMedia
GSM631489/GSM631490/GSM631491	GSE25710	ER	T47D	FullMedia
GSM631492/GSM631493	GSE25710	ER	T47D	FullMedia
GSM1669011	GSE68359	ER	T47D	FullMedia
GSM1669012	GSE68359	ER	T47D	FullMedia
GSM1669013	GSE68359	ER	T47D	FullMedia
GSM1669042	GSE68359	ER	MCF7	FullMedia
GSM1669043	GSE68359	ER	MCF7	FullMedia
GSM1669044	GSE68359	ER	MCF7	FullMedia
GSM1669079	GSE68359	ER	MCF7	E2
GSM1669081	GSE68359	ER	MCF7	E2+PG
GSM1669083	GSE68359	ER	MCF7	E2
GSM1669085	GSE68359	ER	MCF7	E2+PG
GSM1669087	GSE68359	ER	MCF7	E2
GSM1669089	GSE68359	ER	MCF7	E2+PG
GSM1669097	GSE68359	PR	MCF7	E2+PG
GSM1669103	GSE68359	ER	T47D	E2
GSM1669107	GSE68359	ER	T47D	E2
GSM1669117	GSE68359	PR	T47D	E2+PG
GSM1669121	GSE68359	PR	T47D	E2+PG
GSM1669125	GSE68359	PR	T47D	E2+PG
GSM1669134	GSE68359	ER	MCF7 Xenograft	E2+PG
GSM1669135	GSE68359	ER	MCF7 Xenograft	E2+PG
GSM1669136	GSE68359	ER	MCF7 Xenograft	E2+PG
GSM1669137	GSE68359	ER	MCF7 Xenograft	E2+PG
GSM1669138	GSE68359	ER	MCF7 Xenograft	E2+PG
GSM1669139	GSE68359	ER	MCF7 Xenograft	E2+PG
GSM1669140	GSE68357	ER	MCF7 Xenograft	E2
GSM1669141	GSE68357	ER	MCF7 Xenograft	E2
GSM1669142	GSE68357	ER	MCF7 Xenograft	E2
GSM1669143	GSE68357	ER	MCF7 Xenograft	E2
GSM1669144	GSE68357	ER	MCF7 Xenograft	E2
GSM1669145	GSE68357	ER	MCF7 Xenograft	E2
GSM1669156	GSE68357	PR	MCF7 Xenograft	E2
GSM798383	GSE32222	ER	Female Tumor	Responder 1
GSM798384	GSE32222	ER	Female Tumor	Responder 2
GSM798385	GSE32222	ER	Female Tumor	Responder 3
GSM798386	GSE32222	ER	Female Tumor	Responder 4
GSM798387	GSE32222	ER	Female Tumor	Responder 5
GSM798388	GSE32222	ER	Female Tumor	Responder 5
GSM798389	GSE32222	ER	Female Tumor	Responder 6
GSM798391	GSE32222	ER	Female Tumor	Responder 8
GSM798392	GSE32222	ER	Female Tumor	Non-responder 1
GSM798393	GSE32222	ER	Female Tumor	Non-responder 2
GSM798394	GSE32222	ER	Female Tumor	Non-responder 3
GSM798395	GSE32222	ER	Female Tumor	Non-responder 4
GSM798396	GSE32222	ER	Female Tumor	Non-responder 5
GSM798397	GSE32222	ER	Female Tumor	Non-responder 5
GSM798398	GSE32222	ER	Female Tumor	Non-responder 6
GSM798399	GSE32222	ER	Female Tumor	Non-responder 6
GSM798400	GSE32222	ER	Female Tumor	Non-responder 7
GSM1003711	GSE40867	ER	Female Tumor	Good outcome 1
GSM1003714	GSE40867	ER	Female Tumor	Good outcome 2
GSM1003717	GSE40867	ER	Female Tumor	Good outcome 3
GSM1003720	GSE40867	ER	Female Tumor	Good outcome 4
GSM1003723	GSE40867	ER	Female Tumor	Poor outcome 1
GSM1003726	GSE40867	ER	Female Tumor	Poor outcome 2
GSM1003734	GSE40867	ER	Female Tumor	Poor outcome 3
GSM1003737	GSE40867	ER	Female Tumor	Poor outcome 4
GSM1003740	GSE40867	ER	Female Tumor	Poor outcome 5
GSM2154991	GSE81512	GR	MCF7	E2+Dex
GSM2154995	GSE81512	GR	MCF7	E2+Dex
GSM2154997	GSE81512	GATA3	MCF7	E2
GSM2154998	GSE81512	GATA3	MCF7	E2+Dex
GSM986067	GSE40129	GATA3	MCF7	E2
GSM986069	GSE40129	GATA3	MCF7	E2
GSM986071	GSE40129	GATA3	MCF7	E2
GSM986073	GSE40129	GATA3	MCF7	E2
GSM986075	GSE40129	GATA3	MCF7	E2
